# The NLRP3 inflammasome in burns: a novel potential therapeutic target

**DOI:** 10.1093/burnst/tkae020

**Published:** 2024-07-02

**Authors:** Haihong Li, Junhong Zhao, Leilei Cao, Qizhi Luo, Cuiping Zhang, Lei Zhang

**Affiliations:** Department of Burns and Plastic Surgery, Seventh Affiliated Hospital, Sun Yat-sen University, 628 Zhenyuan Road, Guangming District, Shenzhen 518107, Guangdong Province, China; Laboratory of Wound Repair and Dermatologic Surgery, Taihe Hospital, Hubei University of Medicine, 32 South Renmin Road, Shiyan 442000, Hubei Province, China; Department of Burns and Plastic Surgery, Seventh Affiliated Hospital, Sun Yat-sen University, 628 Zhenyuan Road, Guangming District, Shenzhen 518107, Guangdong Province, China; Department of Burns and Plastic Surgery, Seventh Affiliated Hospital, Sun Yat-sen University, 628 Zhenyuan Road, Guangming District, Shenzhen 518107, Guangdong Province, China; Research Center for Tissue Repair and Regeneration affiliated to the Medical Innovation Research Department and Fourth Medical Center of PLA General Hospital, 51 Fucheng Road, Beijing 100048, China; Department of Psychiatry and Clinical Psychology, Seventh Affiliated Hospital, Sun Yat-sen University, 628 Zhenyuan Road, Guangming District, Shenzhen 518107, Guangdong Province, China

**Keywords:** NLRP3 inflammasome, Burns, Non-healing wounds, Complications, Sequelae, Inhibitors

## Abstract

Burns are an underestimated serious injury negatively impacting survivors physically, psychologically and economically, and thus are a considerable public health burden. Despite significant advancements in burn treatment, many burns still do not heal or develop serious complications/sequelae. The nucleotide-binding oligomerization domain-like receptors (NLRs) family pyrin domain-containing 3 (NLRP3) inflammasome is a critical regulator of wound healing, including burn wound healing. A better understanding of the pathophysiological mechanism underlying the healing of burn wounds may help find optimal therapeutic targets to promote the healing of burn wounds, reduce complications/sequelae following burn, and maximize the restoration of structure and function of burn skin. This review aimed to summarize current understanding of the roles and regulatory mechanisms of the NLRP3 inflammasome in burn wound healing, as well as the preclinical studies of the involvement of NLRP3 inhibitors in burn treatment, highlighting the potential application of NLRP3-targeted therapy in burn wounds.

Highlights

- NLRP3 inflammasome activation is beneficial in acute burn wound healing.
- Persistent and dysregulated NLRP3 inflammasome activation is associated with non-healing burn wounds and complications/sequelae following burn.
- Targeted activation of NLRP3 inflammasome signaling in early burn wounds and targeted inhibition of NLRP3 inflammasome signaling in non-healing burn wounds and complications following burn may represent novel therapeutic strategies for burn injuries.

## Background

Burns are among the most common injuries caused by heat, fire, electricity, chemicals or radioactive substances [1–3]. Analysis of the Global Burden of Disease 2017 on thermal injuries in 195 countries and regions revealed 8,991,468 new thermal injuries worldwide, with 120,632 deaths [2]. Globally, the incidence of burns and the age-standardized disability-adjusted life-years due to burns has declined significantly from 1990 to 2017; however, some low-income and middle-income regions, such as East Asia, Southern Latin America, and high-income Asia-Pacific, still showed an increase in incidence [2]. A study on child unintentional injury surveillance in four low- and middle-income countries (Bangladesh, Colombia, Egypt and Pakistan) showed that 17% of children with thermal injury were disabled for >6 weeks and 8% were permanently disabled [4]. According to a Spanish study, the total annual cost of burn patients was as high as US$313 million [5]. Burns are a serious public health problem with high mortality and a negative impact on the physical, psychological and economic well-being of individuals, families and communities [2,6]. Despite great progress made in early fluid resuscitation, infection management, wound management and coverage, nutritional support, organ support, and treatment of comorbidities, many burns still fail to heal or develop severe complications/sequelae [7–10].

The nucleotide*-*binding oligomerization domain (NOD)*-*like receptors (NLRs) family pyrin domain-containing 3 (NLRP3) inflammasome is a key component of the innate immune system, which serves as a platform for the secretion of pro-inflammatory cytokines [11,12]. Many studies have demonstrated the crucial role of NLRP3 in wound healing, including burn wound healing [13–17]. A better understanding of the pathophysiological mechanism underlying the healing of burn wounds can help find optimal therapeutic targets to promote the healing of burn wounds, reduce complications/sequelae following burn, and maximize the restoration of the structure and function of burn skin. This review summarizes the current understanding of the roles and regulatory mechanisms of the NLRP3 inflammasome in burn wound healing, as well as the preclinical studies of NLRP3 inhibitors in burn treatment, highlighting the potential application of NLRP3-targeted therapy in burns.

## Review

### Wound healing and burn wound healing

**Figure 1:**
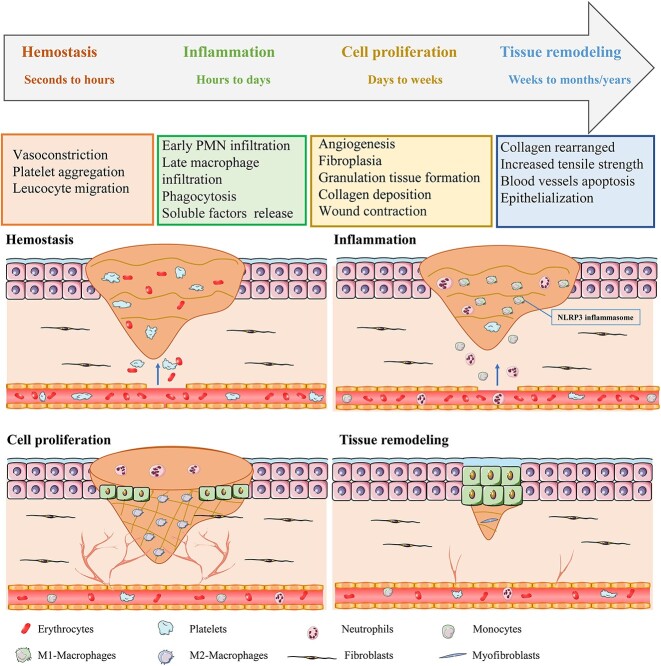
Four phases of physiological wound healing and the major events involved. Physiological wound healing is generally divided into four phases: hemostasis, inflammation, cell proliferation and tissue remodeling. Hemostasis occurs immediately after the injury and lasts for minutes to hours, involving vasoconstriction, platelet aggregation and leucocyte migration at the wound site. Inflammation ensues, lasting hours to days or months, and is characterized by the infiltration of PMNs and monocytes, differentiation of monocytes into pro-inflammatory M1 macrophages, and release of pro-inflammatory cytokines. The NLRP3 inflammasome is activated in macrophages in the inflammation phase. The proliferative phase usually lasts for days to weeks and is characterized by neovascularization, granulation tissue formation, collagen deposition, wound contraction, re-epithelialization and a change in the phenotype of macrophages from a pro-inflammatory M1 to an anti-inflammatory M2 phenotype. The tissue remodeling phase lasts for weeks to months, or even years, and is characterized by collagen rearrangement, granulation tissue degeneration, vascular regression, fibroblast-to-myofibroblast transformation and replacement of type III collagen by type I collagen. *PMNs* polymorphonuclear neutrophils, *NLRP3* nucleotide-binding oligomerization domain-like receptors family pyrin domain-containing 3

Wound healing is a complex, dynamic and highly ordered biological process typically including four phases: hemostasis, inflammation, cell proliferation and tissue remodeling [18,19] (Figure 1). Hemostasis occurs immediately after injury and involves vasoconstriction, followed by the adherence and aggregation of platelets at the wound site. These platelets become amorphous and release chemical signals to activate fibrin [18,19]. Activated fibrin, on the one hand, forms a clot to occlude ruptured blood vessels; on the other hand, it releases bioactive factors that recruit inflammatory cells to the wound site [18,20,21]. The formed fibrin clot serves as a temporary matrix for subsequent wound healing [18,20,21]. Hours after injury, the inflammatory response is activated by pathogen-associated molecular patterns (PAMPs) or danger-associated molecular patterns (DAMPs) [22,23]. Inflammatory cells, primarily polymorphonuclear neutrophils (PMNs) and monocytes, are, in turn, recruited to the wound site to eliminate debris, dead cells and bacteria [7,24]. PMNs are the first cells recruited to injured tissue. They are sentinels of the innate immune system, playing an important role in the early stages of wound healing [24,25]. PMNs defend against pathogens through phagocytosis or release of neutrophil extracellular traps [26]. Once the task of PMNs is complete, they are engulfed and degraded by macrophages as circulating monocytes enter the wound site and differentiate into pro-inflammatory M1-like phenotype [27]. Macrophages not only serve as phagocytic cells, but also promote the release of pro-inflammatory cytokines, such as interleukin-1β (IL-1β), IL-18 and tumor necrosis factor-α (TNF-α), which trigger the innate immune response [27,28]. The inflammation lasts from weeks to months depending on the severity of the injury. A well-orchestrated immune response is critical for a proper healing process, whereas a persistent and dysregulated immune response can negatively influence wound closure and tissue repair [20,29]. After the inflammatory phase, wound healing enters the proliferative phase involving the recruitment and activation of fibroblasts, vascular endothelial cells and keratinocytes to the wound [18,20,21]. The proliferative phase is characterized by replacement of the temporary matrix formed in the hemostasis phase with a connective tissue matrix, neovascularization, granulation tissue formation, collagen deposition, wound contraction and re-epithelialization [18,20,21]. In this phase, the phenotype of macrophages changes from a predominantly pro-inflammatory M1-like phenotype to an anti-inflammatory M2-like phenotype [7,30,31]. The final phase of wound healing is tissue remodeling, which is characterized by collagen rearrangement along tension lines, granulation tissue degradation, vascular regression, fibroblasts transformation to myofibroblasts and replacement of type III collagen by type I collagen in the granulation tissue [7]. Optimal wound healing depends on the availability of sufficient cells from the bone marrow and a balance between pro-inflammatory and anti-inflammatory mediators [7,32]. For successful wound healing, all four phases must occur in the proper sequence and time frame. The desired wound healing outcome is to close the damaged wound and restore the structure and function of the damaged tissues and organs [20,29].

The healing process of burn wounds is similar to that of general wounds; however, it is more complicated and has its own unique characteristics [33,34]. First, the inflammatory cascade is triggered multiple times during burn wound healing. Burn surgery, burn complications such as infection, burn sepsis, and burn-induced acute lung, kidney or liver injury may also trigger an inflammatory response [33,35,36]. Second, the coordinated immune response is distorted in severe burns, leading to a persistent and uncontrolled inflammatory response, also known as a burn-induced hyperinflammatory state [7,25]. The hyperinflammatory state is accompanied by increased release of immature neutrophils and sustained release of pro-inflammatory cytokines [7,25]. Instead of promoting wound healing, the hyperinflammatory state leads to widespread hypermetabolic responses, systemic inflammatory response syndrome, tissue and organ damage and delayed wound healing [7,15]. Third, moderate-to-severe burns are accompanied by severe systemic involvement, such as hypovolemic shock, infection, sepsis and even multiple organ failure, most of which are due to the widespread inflammatory response of the body [35,36]. Fourth, infection occurs not only at the burn site but also in distant organs such as the lungs, urinary tract and blood, which can be fatal. Infection further exacerbates the hypermetabolic state [35]. Fifth, burn-induced hypovolemic shock is mainly due to capillary leakage, resulting in the redistribution of fluid from intravascular to interstitial space [36]. Tissue edema and fluid accumulation result in impaired tissue perfusion and oxygen delivery, eventually leading to dysfunction and injury of the lungs, liver, gastrointestinal tract, heart and even multiple organs. Sixth, burn wounds are associated with profound and persistent hypermetabolic responses [36]. These hypermetabolic responses can persist for up to 24 months, with nearly 50-fold increases in the plasma catecholamines, cortisol and inflammatory factors [7,15]. The hypermetabolic responses after burns are characterized by hyperdynamic cycling, insulin resistance, and increased protein and lipid catabolism. Also, they are associated with increased resting energy expenditure, systemic protein loss, muscle atrophy, poor wound healing, multiple organ dysfunction and even death [7,15].

### NLRP3 inflammasomes

Inflammasomes, which are key sentinels of the innate immune system, are large cytoplasmic multiprotein signaling complexes playing critical roles in defense against pathogens and cell damage [37–39]. They assemble in response to a variety of stimuli, triggering a cascade of downstream responses such as the release of pro-inflammatory cytokines and pyroptotic cell death [37–39]. Several inflammasomes have been described, including NLRP1, NLRP2, NLRP3, NLRP6, NLRP7, NLR-family apoptosis-inhibitory protein 2 (NAIP2), NAIP5, NAIP6, NLR-family caspase activation and recruitment domain (CARD)-containing 4 (NLRC4), absent in melanoma 2 (AIM2) and pyrin [39–41].

**Figure 2:**
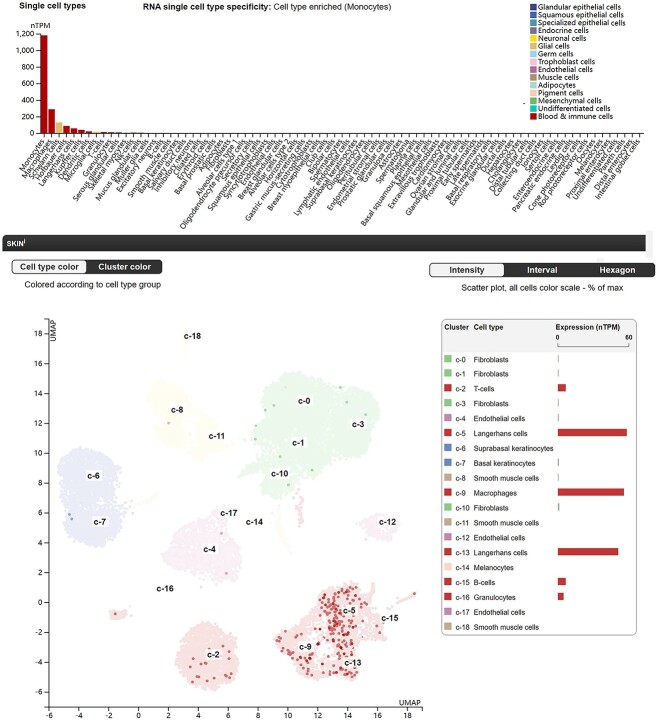
NLRP3-enriched cells. Single-cell RNA in humans showing NLRP3 RNA is enriched in blood and immune cells (upper). Single-cell RNA in human skin shows that NLRP3 is expressed primarily in macrophages and Langerhans cells (lower). Data from the Human Protein Atlas (https://www.proteinatlas.org/). *NLRP3* nucleotide-binding oligomerization domain-like receptors family pyrin domain-containing 3

The NLRP3 inflammasome is the most thoroughly studied inflammasome. It is vital in innate immune defense, homeostasis maintenance, and initiation and propagation of inflammatory responses [38,42,43]. Major reports on the NLRP3 inflammasome involve its expression and role in tissue-resident and infiltrating immune cells, such as monocytes, macrophages and dendritic cells, besides reports on functional NLRP3 activity in parenchymal cells, such as hepatocytes, keratinocytes and podocytes [44–48]. However, Kunte *et al*. showed no NLRP3 inflammasome activity in kidney epithelial cells, even when the NLRP3 variant was expressed in podocytes [49]. The Human Protein Atlas showed that NLRP3 RNA was expressed primarily in blood and immune cells, with some expression in Schwann cells and microglial cells (https://www.proteinatlas.org/ENSG00000162711-NLRP3/single+cell+type) (Figure 2). Single-cell RNA in human skin showed that NLRP3 was expressed primarily in macrophages and Langerhans cells (a type of tissue-resident macrophage), but not in keratinocytes, melanocytes, endothelial cells or smooth muscle cells (https://www.proteinatlas.org/ENSG00000162711-NLRP3/single+cell+type/skin) (Figure 2).

**Figure 3:**
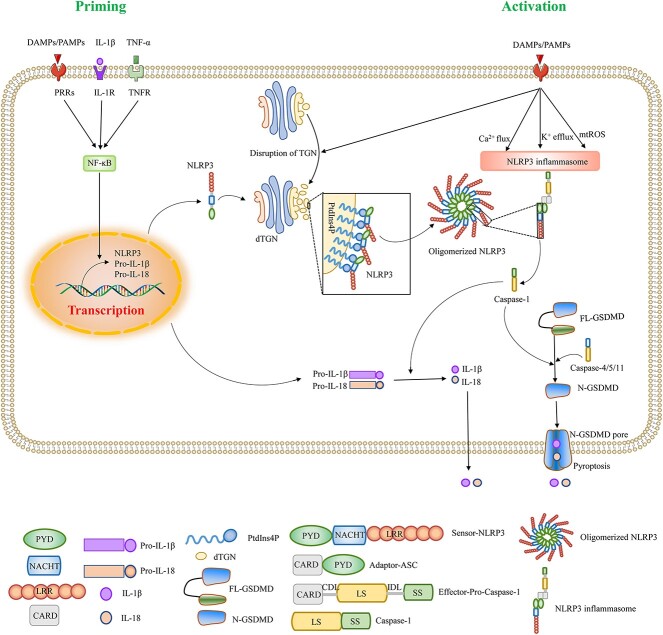
Priming and activation of the NLRP3 inflammasome. Priming of the NLRP3 inflammasome is provided by the binding of PAMPs, DAMPs or cytokines (IL-1β and TNF-α) to their respective receptors. Upon binding of the ligand to the receptor, NF-κB is activated and translocated to the nucleus, where it upregulates NLRP3, pro-IL-1β and pro-IL-18. The NLRP3 inflammasome is activated by a variety of PAMPs or DAMPs that induce multiple signaling events such as disassembly of the trans-Golgi network, K^+^ efflux, Ca^2+^ flux and mtROS production. The dispersed TGNs serve as scaffolds for NLRP3 aggregation and oligomerization. Oligomerized NLRP3 recruits ASCs, and multiple ASCs aggregate to form ASC specks, which then recruit pro-caspase-1. Inactive pro-caspase-1 forms active caspase-1 by autoproteolysis. On the one hand, caspase-1 cleaves pro-IL-1β and pro-IL-18 into mature bioactive IL-1β and IL-18, respectively; on the other hand, caspase-1 cleaves GSDMD, and N-GSDMD forms a membrane pore, triggering pyroptosis. *NLRP3* nucleotide-binding oligomerization domain-like receptors family pyrin domain-containing 3, *TNF-α* tumor necrosis factor-α, *TNFR* tumor necrosis factor receptor, *IL-1R* IL-1 receptor, *PAMPs* pathogen-associated molecular patterns, *DAMPs* damage-associated molecular patterns, *NF-κB* nuclear factor-κB, *IL-1β* interleukin-1β, *IL-18* interleukin-18, *pro-IL-1β* interleukin-1β precursor, *pro-IL-18* interleukin-18 precursor, *mtROS* mitochondrial reactive oxygen species, *TGN* trans-Golgi network, *dTGN* dispersed TGNs, *PtdIns4P* phosphatidylinositol-4-phosphate, *PYD* pyrin domain, *NACHT* nucleotide-binding oligomerization domain, *LRR* leucine-rich repeat, *CARD* caspase-recruitment domain, *LS* large catalytic subunit domain, *SS* small catalytic subunit domain, *CDL* CARD domain linker, *IDL* interdomain linker, *GSDMD* Gasdermin D, *N-GSDMD* N-terminus of GSDMD

The NLRP3 inflammasome is composed of a sensor (NLRP3), an adapter (apoptosis-associated speck-like protein containing a caspase recruitment domain; ASC) and an effector (pro-caspase-1) [42,50] (Figure 3). NLRP3 is a tripartite protein consisting of an N-terminal pyrin domain (PYD), a central nucleotide-binding and oligomerization domain (NOD; also referred to as NACHT), and a C-terminal leucine-rich repeat domain [50]. The ASC is a bipartite protein composed of an N-terminal PYD and a caspase-recruitment domain (CARD) [50,51]. Pro-caspase-1 contains three domains: an N-terminal CARD, a central large catalytic subunit domain (p20, LS), and a C-terminal small catalytic subunit domain (p10, SS). The CARD is linked with the large catalytic subunit domain by a CARD domain linker (CDL), and the large subunit is linked with the small subunit by the interdomain linker (IDL) [11,50] (Figure 3).

NLRP3 inflammasome activation is a strictly regulated process that typically requires both priming and activation signals [11,50]. Priming of the NLRP3 inflammasome occurs through the binding of PAMPs or DAMPs to pattern-recognition receptors such as Toll-like receptors (TLRs), or through the binding of cytokines such as IL-1β and TNF-α to their respective receptors [11,50,52]. Upon ligand binding to the receptor, nuclear factor kappa B (NF-κB) is activated and translocated to the nucleus, where it upregulates the transcription of NLRP3, pro-IL-1β and pro-IL-18 [11,50]. In macrophages, pro-IL-1β is not constitutively expressed under resting conditions and NLRP3 expression is insufficient to initiate inflammasome activation [53,54]. Thus, activating the NLRP3 inflammasome in macrophages requires priming and activation steps [53,54]. In contrast, human monocytes can form the NLRP3 inflammasome in the absence of priming [55].

The priming step licenses the cell, whereas the activation step induces assembly and full activation of the NLRP3 inflammasome in the cell [50]. The NLRP3 inflammasome can be activated by a wide variety of exogenous PAMPs associated with microbial-derived substances, and endogenous DAMPs associated with cell-derived substances [54,56]. These chemically and structurally unrelated stimuli trigger a common cellular event leading to cytosolic protein NLRP3 activation [42]. How NLRP3 responds to various signaling events and initiates the assembly and activation of the NLRP3 inflammasome has been controversial [42]. Mitochondrial dysfunction, lysosomal damage, reactive oxygen species and ion flux have all been proposed to trigger NLRP3 activation [11,57–59]. However, these proposed mechanisms are important for NLRP3 activation in response to some stimuli, but not all [11].

Chen and Chen found that different NLRP3 stimuli led to the disassembly of the trans-Golgi network (TGN), and then the dispersed TGN (dTGN) acted as a scaffold for NLRP3 aggregation and oligomerization and subsequent activation [42]. The NLRP3 inflammasome activation model is similar to the “guard model” in plants [11]. Oligomerized NLRP3 on dTGN recruits ASC through the interaction of the PYD of NLRP3 with the PYD of ASC [42]. The binding of NIMA-associated kinase 7, a serine/threonine kinase, to the leucine-rich repeat domain of NLRP3 is necessary for the formation of NLRP3 and adapter ASC complexes, ASC specks, and caspase-1 activation [60]. Multiple ASCs aggregate to form ASC specks, and then the assembled ASC specks recruit pro-caspase-1 through CARD–CARD interactions [42,56]. Inactive pro-caspase-1 is converted into a catalytic active form by the autoproteolytic removal of CARD and IDL [11,50]. Once pro-caspase-1 clusters on ASC, pro-caspase-1 self-cleaves at the IDL to generate a complex of p33 (comprising the CARD and p20) and p10, which is bound to ASC but still has proteolytic activity [11,50]. Subsequently, pro-caspase-1 further self-cleaves at the CDL and releases the unstable p20–p10 tetramer from the ASC–pro-caspase-1 complex, leading to the formation of the caspase-1 active form, the tetramer of two p20/p10 heterodimers [11,50]. Upon activation, caspase-1, on the one hand, cleaves the potent pro-inflammatory cytokines pro-IL-1β and pro-IL-18 into their mature and bioactive forms IL-1β and IL-18, which are then released from the cells. On the other hand, caspase-1 cleaves gasdermin D (GSDMD), and the cleaved N-terminus of GSDMD (N-GSDMD) forms irregular membrane pores, triggering pyroptosis [11,50] (Figure 3). NLRP3/caspase-1-dependent IL-1β/IL-18 release or NLRP3/caspase-1-dependent/GSDMD-mediated pyroptosis is crucial in inflammatory disease, autoimmune diseases, tissue injuries and other diseases [61–64].

### NLRP3 inflammasomes in burns

The NLRP3 inflammasome is involved in coagulation, inflammation, cell proliferation, angiogenesis and tissue remodeling [15,65,66]. Activation of the NLRP3 inflammasome is a double-edged sword in burn wound healing [13,14,67,68]. NLRP3 inflammasome activation is beneficial in early burn wound healing, whereas prolonged and dysregulated NLRP3 inflammasome activation leads to adverse outcomes such as non-healing burn wounds and burn complications/sequelae [13,67,68]. Therefore, the differential control of NLRP3 inflammasome activation is required for optimal burn wound healing.

#### Beneficial role of NLRP3 inflammasome in early burn wound healing

Once burns compromise the skin barrier, an inflammatory response primarily mediated by the NLRP3 inflammasome is triggered [13,69]. In human burn skin with a mean total body surface area (TBSA) of 30.3% ± 3.29%, the gene and protein expression of NLRP3 and IL-1β (a by-product of NLRP3 activation) increased 0–2 days after burn, and the gene expression of IL-18 (a by-product of NLRP3 activation) increased 3–6 days after burn [13]. The results obtained using both mouse and rat burn models were similar to those obtained using human burn wounds [13,70]. In a 30–35% TBSA third-degree mouse burn model, the protein expression ratio of cleaved to pro-caspase-1 and cleaved to pro-IL-1β increased 0–2 days after burn compared with that in the normal skin [13]. In a 30% TBSA deep second-degree burn rat model, burn injury induced marked activation of the NLRP3 inflammasome and cleavage of IL-1β in macrophages in stasis zones [70]. In a 30% TBSA third-degree burn mouse model, NLRP3^−/−^ burn mice had fewer NLRP3-positive cells in the dermis compared with wild-type (WT) burn mice [13]. Compared with the WT burn mice, NLRP3^−/−^ burn mice exhibited impaired wound healing, reduced macrophage infiltration, and decreased expression of pro-inflammatory cytokines, chemokines and growth factors, but the early presence of and increased anti-inflammatory macrophages [13]. Blocking the activation of caspase-1, the end product of the NLRP3 inflammasome pathway, with *N*-acetyl-tyrosyl-valyl-alanyl-aspartyl chloromethyl ketone resulted in significantly increased mortality in burn-injured mice [71]. Therefore, the NLRP3 inflammasome plays a beneficial role in early burn wound healing [13,14,72]. The NLRP3 inflammasome enhances the defense of the innate immune system by modulating inflammation and macrophage polarization [13,73].

#### Persistent and dysregulated NLRP3 inflammasome activation in non-healing burn wounds and complications/sequelae following burn

Non-healing/chronic burn wounds and complications/sequelae following burn, such as burn sepsis, burn-induced kidney/lung/liver injury, keloids and scars, are characterized by persistent and dysregulated inflammation [15,67,74].

##### Non-healing burn wounds

One of the hallmarks of cell proliferation is the transition of macrophages from an M1 pro-inflammatory phenotype to an M2 anti-inflammatory phenotype [31,73]. The impairment of this transition leads to increased numbers of M1 pro-inflammatory macrophages in wounds [31,73]. M1 macrophages exhibit strong NLRP3 inflammasome activity, creating a feedback loop that keeps the M1 macrophages in an inflamed state [31]. High expression of NLRP-3, caspase-1, IL-1β and IL-18 was observed in macrophages isolated from wounds of patients with type 2 diabetes mellitus whose wounds lasted for at least 3 months [75]. In a diabetic db/db mouse model of full-thickness skin excision wounds, IL-1β and IL-18 expression in macrophages remained elevated 10 days after wounding [75]. Sustained activation of the NLRP3 inflammasome resulted in delayed corneal wound healing and impaired nerve regeneration in WT mice with diabetes, whereas corneal epithelial wound closure and neurogenesis were significantly accelerated in NLRP3^−/−^ mice with diabetes [76]. These studies suggested that the NLRP3 inflammasome was a key regulator of chronic inflammatory response in wounds of humans and mice with diabetes. In injured patients, including burn patients, marked pathophysiological changes and persistent chronic inflammatory state disrupt the timely progression of wound healing, leading to impaired wound closure and ultimately, chronic wound and delayed recovery [20,29,31,77].

##### Burn sepsis

Autopsy studies have shown that ~61% of deaths in burn patients were attributable to infectious complications [7,78]. Sepsis was the leading cause of death in burn patients [35,68]. The NLRP3 inflammasome orchestrates burn-induced, inflammatory-driven pathophysiologic processes [13]. A study evaluating NLRP3 inflammasome gene expression among healthy controls and non-septic and septic adult burn patients in the early (0–11 days after burn) and later (≥12 days after burn) stages showed that non-septic burn patients had increased gene expression of IL-1β, IL-18 and NLRP3 compared with healthy controls in the early stages, but the expression of NLRP3 inflammasome components returned to baseline or control levels in the later stages [68]. However, in the later post-burn period, NLRP3 inflammasome gene expression was significantly higher in septic burn patients than in controls and non-septic burn patients [68]. The mice were subjected to a two-hit model of 25–30% TBSA scald burn, followed by *Pseudomonas aeruginosa* wound infection 72 h after burn, to further explore the roles and mechanisms of the NLRP3 inflammasome in burn sepsis. The results showed that NLRP3^−/−^ mice with burn sepsis had 30% increased survival and bacterial clearance at the injury site compared with WT mice with burn sepsis. Also, these mice had more macrophage and neutrophil infiltration at the injury site and adipose tissue at 12 h after infection, and subsequently in lymphoid organs and liver [68]. Collectively, the persistence and dysregulation of NLRP3 inflammasome activation are common features of burn sepsis and are associated with poor prognosis. Targeted inhibition of the NLRP3 inflammasome heralds a new therapeutic intervention to improve burn sepsis and its poor prognosis [13,68].

##### Burn-induced acute lung injury and acute respiratory distress syndrome

Acute lung injury (ALI) and acute respiratory distress syndrome (ARDS) may occur due to inhalation injury or may be mediated by post-burn inflammatory response, especially when burns are accompanied by shock, infection, sepsis or delayed resuscitation [79]. ALI and ARDS are the most serious complications associated with high mortality [80]. In addition, inhalation injury is an independent predictor of mortality in burn patients [79]. Therefore, early and optimal ALI and ARDS intervention is critical to improve the prognosis of burn patients.

The lung wet-to-dry weight ratio, lung NLRP3 and caspase-1 protein expression, and serum TNF-α, IL-8, IL-1β and IL-18 concentrations all significantly increased in rat models of ALI induced by 30% TBSA third-degree burns compared with rats in the sham group [81,82]. Another study showed that the serum myeloperoxidase activity and malondialdehyde content, as well as NF-κB and matrix metalloproteinase-9 protein expression were also significantly higher in the lungs of rats in the burn group compared with those in the sham group [81]. In another ALI model of rats with 40% TBSA third-degree burns, the rats in the burn group showed diffuse exudative changes and obvious pathological changes, such as pulmonary interstitial congestion, edema, hemorrhage, alveolar capillary wall rupture and extensive inflammatory cell infiltration, 12 and 24 h after burn [83]. The expression levels of NLRP3 and caspase-1 proteins, and the concentrations of IL-18 and IL-1β were significantly higher in the lung tissue of rats in the burn group compared with those in the sham group [83]. In a burn rat model of simulated aviation medical evacuation, rats exposed to 30% TBSA third-degree burns plus normoxia had significantly increased release levels of IL-1β, IL-6 and mitochondrial deoxyribonucleic acid in serum, and significantly increased expression of NLRP3 inflammasome, malondialdehyde content and myeloperoxidase activity in the lung compared with normal rats [84]. Burn plus hypobaric hypoxia exposure further exacerbated this situation [84]. All these studies suggest the involvement of the NLRP3 inflammasome in the pathogenesis of ALI and ARDS after burn. Inhibition of the NLRP3 inflammasome protects against ALI and ARDS induced by burn injury [81–84].

##### Hypertrophic scarring and keloids

Burn patients are prone to devastating sequelae, such as hypertrophic scarring and keloids, even after burn healing, resulting in altered skin appearance and dysfunction [85]. The incidence of hypertrophic scar after burns is as high as 91% [86]. A study of human burn tissue showed that the NLRP3 inflammasome was activated in burn skin 0–2 days after burn and its levels returned to baseline by 7–10 days after burn [85]. However, the expression of cleaved caspase-1, IL-1β and IL-18 was upregulated in human keloids 7–10 days after burn compared with burn and normal skin, suggesting NLRP3-mediated inflammation in keloids and possibly contributing to a persistent inflammatory state [85]. Persistent inflammation and immune cell infiltration lead to increased fibroblast activity, excessive collagen synthesis, insufficient matrix degradation and remodeling, and persistent extracellular matrix deposition [85].

Low-grade metabolic inflammation plays an essential role in the pathogenesis of scarring [86]. Pro-inflammatory M1 macrophages favor glycolysis [87]. Increased glucose influx and glycolysis regulate the priming and activation of NLRP3 [85]. The expression of glucose transporter 1 (Glut1) and glycolytic genes such as hexokinase, phosphofructokinase, pyruvate dehydrogenase kinase 1 and pyruvate kinase M2 was upregulated in keloids and burn skin compared with normal skin, indicating enhanced glycolysis [85]. The expression of Glut1 was significantly higher in keloid burn skin compared with non-keloid burn skin [85]. The fibroblasts isolated from keloids, hypertrophic scars and fibrosis had higher glucose influx and lactate production compared with normal fibroblasts [85].

### Drugs targeting the NLRP3 inflammasome in burns

Healing of chronic burn wounds and management of complications/sequelae following burn are challenging public health problems worldwide. Despite the important roles of the NLRP3 inflammasome in burns and complications/sequelae following burn, the available treatments remain limited [88]. Several NLRP3 inflammasome activation inhibitors, including MCC950, 3,4-methylenedioxy-β-nitrostyrene (MNS), glyburide and Bay 11–7082, have been used to target the NLRP3 inflammasome in burn treatment in animals [70,82,89,90]. The development of molecules and drugs directly targeting NLRP3 or indirectly targeting inflammasome components or related signaling events heralds a new therapeutic intervention to improve the adverse outcomes of burns and opens a new chapter in burn treatment [91,92].

#### MCC950

MCC950, a diarylsulfonylurea-containing compound, is a potent and selective small molecule that inhibits NLRP3 activation [93,94]. MCC950 specifically inhibited NLRP3 activation, without affecting NLRC4, NLRP1, AIM2, TLR signaling or NLRP3 priming, in mouse bone marrow-derived macrophages and human monocyte-derived macrophages. Also, it blocked NLRP3-induced ASC oligomerization but not K^+^ efflux, Ca^2+^ flux or NLRP3–ASC interaction [95]. MCC950 did not lead to a complete block of IL-1β production during infection, thereby maintaining the anti-infection response [95].

In streptozotocin (STZ)-induced male C57BL/6 mice with diabetes, the subconjunctival injection of MCC950 promoted diabetic corneal wound healing, accelerated re-epithelialization, improved corneal sensation and nerve fiber density, and reduced corneal NLRP3 and IL-1β mRNA transcription and IL-1β protein levels [96]. MCC950 pretreatment in mice reduced the expression of NLRP3, ASC, IL-1β and cleaved caspase-1; inhibited the expression of IL-1β, IL-6, IL-8, IL-18 and TNF-α; and decreased malondialdehyde content and lactate dehydrogenase activity in canine corneal stromal cells infected with *Streptococcus pseudointermedius* [97]. This suggested that MCC950 attenuated inflammatory responses. However, these findings were inconsistent with the results of some studies. Lee *et al*. showed no significant differences in wound closure, re-epithelialization or angiogenesis in WT mice and genetically obese Ob/Ob mice treated with MCC950 compared with control mice in a mouse model of full-thickness excisional wounds [91]. No significant differences in the number of macrophages or polarization were observed in wounds of Ob/Ob mice between control and MCC950-treated groups [91]. Deuis *et al*. showed that NLRP3^−/−^ mice, caspase-1^−/−^ mice and mice administered with MCC950 exhibited normal development of mechanical and thermal allodynia in a superficial burn model of left hind paw, suggesting that the NLRP3 inflammasome played a limited role in burn-induced pain [90]. Further studies are needed to demonstrate the exact potential of MCC950.

#### Glyburide

Glyburide is a clinically approved second-generation sulfonylurea that reduces blood glucose concentrations and glycohemoglobin levels [98]. In addition, it also acts as an NLRP3 activation inhibitor [13,99]. Vinaik *et al.* showed that daily glyburide-treated WT burn mice (0–7 days after burn) exhibited decreased expression of IL-1β and IL-18, reduced dermal collagen deposition, decreased macrophage infiltration and increased number of M2 macrophages compared with untreated burn WT mice [13]. The timing of glyburide administration was critical for wound healing [13]. Delayed glyburide-treated (3–7 days after burn) burn WT mice had more collagen deposition and macrophage infiltration compared with daily glyburide-treated burn WT mice [13]. Delayed glyburide-treated burn WT mice mimicked untreated burn WT mice in terms of wound healing and histological features, whereas the daily glyburide-treated burn WT mice resembled burn NLRP3^−/−^ mice, both with impaired wound healing [13]. *In vitro*, glyburide prevented lipopolysaccharides + adenosine triphosphate (ATP)-induced caspase-1 activation, IL-1β secretion and macrophage death [100]. However, glyburide was not a specific inhibitor of the NLRP3 inflammasome [13,100]. It also inhibited the P2X7 receptor and the ATP binding cassette subfamily A member 1 and prevented microbial ligand-, DAMP- and crystal-induced IL-1β secretion [100].

#### Bay 11–7082

Bay 11–7082, a previously identified NF-κB inhibitor, selectively and irreversibly inhibits TNF-α-induced nuclear factor of kappa light polypeptide gene enhancer in B-cells inhibitor-alpha (IκBα) phosphorylation [101,102]. Recent studies showed that Bay 11–7082 was also a selective inhibitor of the NLRP3 inflammasome that inhibited IL-1β secretion, ATPase activity in the NACHT domain of NLRP3, GSDMD pore formation and inflammasome-mediated pyroptosis [103,104]. The histopathology showed that an intraperitoneal injection of 15 μmol/kg BAY11–7082 attenuated burn-induced ALI and decreased myeloperoxidase activity, inflammatory cytokine levels in lung tissue and protein concentrations in bronchoalveolar lavage fluid in a 30% TBSA third-degree burn rat model [82]. BAY 11–7082 blocked the activation of the NLRP3 inflammasome, reduced IL-1β and IL-18 levels, and improved impaired healing patterns in the skin incision wound model of genetically diabetic (db^+^/db^+^) mice [105].

#### MNS

MNS, a spleen tyrosine kinase kinase inhibitor, was previously reported to inhibit platelet aggregation and tumor cell growth, and induce apoptosis [106,107]. It inhibits NLRP3 inflammasome activity by blocking inflammasome assembly [108,109]. Further study showed that MNS specifically blocked NLRP3-mediated ASC speck formation and oligomerization without blocking NLRP3 agonist-induced potassium efflux [108]. MNS treatment inhibited burn-induced NLRP3 inflammasome activation, reduced the production of inflammatory cytokines and neutrophil infiltration, improved burn wound blood perfusion, promoted burn wound healing, and shortened wound re-epithelialization time in a 30% TBSA deep second-degree burn rat model [70].

#### Other drugs

Other drugs shown to improve complications/sequelae following burn by inhibiting NLRP3 inflammasome activation include artemisinin, apelin, sodium butyrate (NaB), β-hydroxybutyric acid (HBA), etc. [89,92,109]. *In vitro*, artemisinin down-regulated the protein levels of NLRP3 and caspase-1 and inhibited the increase in IL-1β and IL-18 mRNA expression in burn sepsis serum-stimulated macrophages. *In vivo*, artemisinin reduced inflammatory cytokine production in serum, levels of adhesion molecules and neutrophil infiltration in the lung and heart, and mortality in mice with burn sepsis [109]. Apelin treatment inhibited NLRP3 inflammasome activity, reduced plasma inflammatory cytokine levels, and reduced mortality in male rats with 40% TBSA third-degree burns [89]. In C57BL/6 mice with unilateral central corneal alkali burns, NaB- and HBA-treated corneas remained transparent and had reduced mRNA levels of NLRP3, caspase-1 and IL-1β compared with vehicle-treated burned corneas [92]. Blocking the NLRP3 pathway reduced inflammation and improved corneal clarity [92].

## Conclusions

NLRP3 inflammasome activation plays a beneficial role in physiological/acute burn wound healing. Also, persistent and dysregulated NLRP3 inflammasome activation is associated with non-healing burn wounds and complications/sequelae following burn. Therefore, the targeted activation of NLRP3 inflammasome signaling in early burn wounds, and the targeted inhibition of NLRP3 inflammasome signaling in non-healing burn wounds and complications following burn, may represent novel therapeutic strategies for burn injuries.

## References

[1] Chen Z , Zhang M, Xie S, Zhang X, Tang S, Zhang C, et al. Global burden of thermal burns, 1990-2017: unbalanced distributions and temporal trends assessed from the global burden of disease study 2017. Burns: journal of the International Society for Burn Injuries. 2022;48:915–25. 34916089 10.1016/j.burns.2021.08.002

[2] James SL , Lucchesi LR, Bisignano C, Castle CD, Dingels ZV, Fox JT, et al. Epidemiology of injuries from fire, heat and hot substances: global, regional and national morbidity and mortality estimates from the global burden of disease 2017 study. Injury prevention: journal of the International Society for Child and Adolescent Injury Prevention. 2020;26:i36–45. 31857422 10.1136/injuryprev-2019-043299 PMC7571358

[3] Schaefer TJ , Tannan SC. Thermal Burns. In: Aboubakr S, Abu-Ghosh A, Ackley WB (eds.), StatPearls. Treasure Island (FL): StatPearls Publishing Copyright © 2023. St. Petersburg, Florida, United States: StatPearls Publishing LLC, 2023.

[4] Hyder AA , Sugerman DE, Puvanachandra P, Razzak J, El-Sayed H, Isaza A, et al. Global childhood unintentional injury surveillance in four cities in developing countries: a pilot study. Bull World Health Organ. 2009;87:345–52. 19551252 10.2471/BLT.08.055798 PMC2678776

[5] Sanchez JL , Bastida JL, Martínez MM, Moreno JM, Chamorro JJ. Socio-economic cost and health-related quality of life of burn victims in Spain. Burns: journal of the International Society for Burn Injuries. 2008;34:975–81. 18472221 10.1016/j.burns.2007.12.011

[6] Kaddoura I , Abu-Sittah G, Ibrahim A, Karamanoukian R, Papazian N. Burn injury: review of pathophysiology and therapeutic modalities in major burns. Annals of burns and fire disasters. 2017;30:95–102. 29021720 PMC5627559

[7] Jeschke MG , van Baar ME, Choudhry MA, Chung KK, Gibran NS, Logsetty S. Burn injury. Nat Rev Dis Prim. 2020;6:11. 32054846 10.1038/s41572-020-0145-5 PMC7224101

[8] Oryan A , Alemzadeh E, Moshiri A. Burn wound healing: present concepts, treatment strategies and future directions. J Wound Care. 2017;26:5–19. 28103165 10.12968/jowc.2017.26.1.5

[9] Ashouri S . An introduction to burns. Phys Med Rehabil Clin N Am. 2022;33:871–83. 36243477 10.1016/j.pmr.2022.07.001

[10] Cancio LC . Topical antimicrobial agents for burn wound care: history and current status. Surg Infect. 2021;22:3–11. 10.1089/sur.2020.368 33124942

[11] Kelley N , Jeltema D, Duan Y, He Y. The NLRP3 Inflammasome: an overview of mechanisms of activation and regulation. Int J Mol Sci. 2019;20:3328. 10.3390/ijms20133328. PMC6651423 31284572

[12] He Y , Hara H, Núñez G. Mechanism and regulation of NLRP3 Inflammasome activation. Trends Biochem Sci. 2016;41:1012–21. 27669650 10.1016/j.tibs.2016.09.002 PMC5123939

[13] Vinaik R , Abdullahi A, Barayan D, Jeschke MG. NLRP3 inflammasome activity is required for wound healing after burns. Translational research: the journal of laboratory and clinical medicine. 2020;217:47. 31843468 10.1016/j.trsl.2019.11.002 PMC7036017

[14] Vinaik R , Stanojcic M, Jeschke MG. NLRP3 Inflammasome modulates post-burn lipolysis and hepatic fat infiltration via fatty acid synthase. Sci Rep. 2018;8:15197. 30315247 10.1038/s41598-018-33486-9 PMC6185951

[15] Vinaik R , Barayan D, Jeschke MG. NLRP3 Inflammasome in inflammation and metabolism: identifying novel roles in Postburn adipose dysfunction. Endocrinology. 2020;161:bqaa116. 10.1210/endocr/bqaa116. PMC7426001 32790834

[16] Huang W , Jiao J, Liu J, Huang M, Hu Y, Ran W, et al. MFG-E8 accelerates wound healing in diabetes by regulating ``NLRP3 inflammasome-neutrophil extracellular traps'' axis. Cell death discovery. 2020;6:84. 32963812 10.1038/s41420-020-00318-7 PMC7484765

[17] Yang H , Zhang Y, Du Z, Wu T, Yang C. Hair follicle mesenchymal stem cell exosomal lncRNA H19 inhibited NLRP3 pyroptosis to promote diabetic mouse skin wound healing. Aging. 2023;15:791–809. 36787444 10.18632/aging.204513 PMC9970314

[18] Sorg H , Tilkorn DJ, Hager S, Hauser J, Mirastschijski U. Skin wound healing: an update on the current knowledge and concepts. European surgical research Europaische chirurgische Forschung Recherches chirurgicales europeennes. 2017;58:81–94. 27974711 10.1159/000454919

[19] Rodrigues M , Kosaric N, Bonham CA, Gurtner GC. Wound healing: a cellular perspective. Physiol Rev. 2019;99:665–706. 30475656 10.1152/physrev.00067.2017 PMC6442927

[20] Martin P , Nunan R. Cellular and molecular mechanisms of repair in acute and chronic wound healing. Br J Dermatol. 2015;173:370–8. 26175283 10.1111/bjd.13954 PMC4671308

[21] Wilkinson HN , Hardman MJ. Wound healing: cellular mechanisms and pathological outcomes. Open Biol. 2020;10:200223. 10.1098/rsob.200223. 32993416 PMC7536089

[22] D'Arpa P , Leung KP. Toll-like receptor Signaling in burn wound healing and scarring. Adv Wound Care (New Rochelle). 2017;6:330–43. 29062590 10.1089/wound.2017.0733 PMC5649422

[23] Rani M , Nicholson SE, Zhang Q, Schwacha MG. Damage-associated molecular patterns (DAMPs) released after burn are associated with inflammation and monocyte activation. Burns: journal of the International Society for Burn Injuries. 2017;43:297–303. 28341255 10.1016/j.burns.2016.10.001 PMC5373089

[24] Wilgus TA , Roy S, McDaniel JC. Neutrophils and wound repair: positive actions and negative reactions. Adv Wound Care (New Rochelle). 2013;2:379–88. 24527354 10.1089/wound.2012.0383 PMC3763227

[25] Mulder PPG , Vlig M, Boekema B, Stoop MM, Pijpe A, van Zuijlen PPM, et al. Persistent systemic inflammation in patients with severe burn injury is accompanied by influx of immature neutrophils and shifts in T cell subsets and cytokine profiles. Front Immunol. 2020;11:11 621222. 33584717 10.3389/fimmu.2020.621222 PMC7879574

[26] DeLeo FR , Allen LH. Phagocytosis and neutrophil extracellular traps. Faculty reviews. 2020;9:25. 33659957 10.12703/r/9-25 PMC7886055

[27] Silva MT . Macrophage phagocytosis of neutrophils at inflammatory/infectious foci: a cooperative mechanism in the control of infection and infectious inflammation. J Leukoc Biol. 2011;89:675–83. 21169518 10.1189/jlb.0910536

[28] Li M , Hou Q, Zhong L, Zhao Y, Fu X. Macrophage related chronic inflammation in non-healing wounds. Front Immunol. 2021;12:12 681710. 10.3389/fimmu.2021.681710 PMC8242337 34220830

[29] Raziyeva K , Kim Y, Zharkinbekov Z, Kassymbek K, Jimi S, Saparov A. Immunology of acute and chronic wound healing. Biomol Ther. 2021;11:700. 10.3390/biom11050700. PMC8150999 34066746

[30] Evers LH , Bhavsar D, Mailänder P. The biology of burn injury. Exp Dermatol. 2010;19:777–83. 20629737 10.1111/j.1600-0625.2010.01105.x

[31] Boniakowski AE , Kimball AS, Jacobs BN, Kunkel SL, Gallagher KA. Macrophage-mediated inflammation in normal and diabetic wound healing. J Immunol (Baltimore, Md: 1950). 2017;199:17–24. 28630109 10.4049/jimmunol.1700223

[32] Muthu K , He LK, Melstrom K, Szilagyi A, Gamelli RL, Shankar R. Perturbed bone marrow monocyte development following burn injury and sepsis promote hyporesponsive monocytes. Journal of burn care & research: official publication of the American Burn Association. 2008;29:12–21. 18182893 10.1097/BCR.0b013e31815fa499

[33] Rowan MP , Cancio LC, Elster EA, Burmeister DM, Rose LF, Natesan S, et al. Burn wound healing and treatment: review and advancements. Critical care. London, England: BioMed Central (England), 2015;19:243. 26067660 10.1186/s13054-015-0961-2 PMC4464872

[34] Wang Y , Beekman J, Hew J, Jackson S, Issler-Fisher AC, Parungao R, et al. Burn injury: challenges and advances in burn wound healing, infection, pain and scarring. Adv Drug Deliv Rev. 2018;123:3. 28941987 10.1016/j.addr.2017.09.018

[35] Church D , Elsayed S, Reid O, Winston B, Lindsay R. Burn wound infections. Clin Microbiol Rev. 2006;19:403–34. 16614255 10.1128/CMR.19.2.403-434.2006 PMC1471990

[36] Markiewicz-Gospodarek A , Kozioł M, Tobiasz M, Baj J, Radzikowska-Büchner E, Przekora A. Burn wound healing: clinical complications, medical care, treatment, and dressing types: the current state of knowledge for clinical practice. Int J Environ Res Public Health. 2022;19:1338. 10.3390/ijerph19031338. PMC8834952 35162360

[37] Barnett KC , Li S, Liang K, Ting JP. A 360° view of the inflammasome: mechanisms of activation, cell death, and diseases. Cell. 2023;186:2288–312. 37236155 10.1016/j.cell.2023.04.025 PMC10228754

[38] Li Y , Huang H, Liu B, Zhang Y, Pan X, Yu XY, et al. Inflammasomes as therapeutic targets in human diseases. Signal transduction and targeted therapy. 2021;6:247. 34210954 10.1038/s41392-021-00650-z PMC8249422

[39] Christgen S , Place DE, Kanneganti TD. Toward targeting inflammasomes: insights into their regulation and activation. Cell Res. 2020;30:315–27. 32152420 10.1038/s41422-020-0295-8 PMC7118104

[40] Zhao Y , Shi J, Shi X, Wang Y, Wang F, Shao F. Genetic functions of the NAIP family of inflammasome receptors for bacterial ligands in mice. J Exp Med. 2016;213:647–56. 27114610 10.1084/jem.20160006 PMC4854738

[41] Ranson N , Kunde D, Eri R. Regulation and sensing of Inflammasomes and their impact on intestinal health. Int J Mol Sci. 2017;18:2379. 10.3390/ijms18112379. PMC5713348 29120406

[42] Chen J , Chen ZJ. PtdIns4P on dispersed trans-Golgi network mediates NLRP3 inflammasome activation. Nature. 2018;564:71–6. 30487600 10.1038/s41586-018-0761-3 PMC9402428

[43] Huang Y , Xu W, Zhou R. NLRP3 inflammasome activation and cell death. Cellular & molecular immunology. 2021;18:2114–27. 34321623 10.1038/s41423-021-00740-6 PMC8429580

[44] Blevins HM , Xu Y, Biby S, Zhang S. The NLRP3 Inflammasome pathway: a review of mechanisms and inhibitors for the treatment of inflammatory diseases. Front Aging Neurosci. 2022;14:14 879021. 10.3389/fnagi.2022.879021 PMC9226403 35754962

[45] Dai X , Tohyama M, Murakami M, Sayama K. Epidermal keratinocytes sense dsRNA via the NLRP3 inflammasome, mediating interleukin (IL)-1β and IL-18 release. Exp Dermatol. 2017;26:904–11. 28266737 10.1111/exd.13334

[46] Xiao Y , Zhao C, Tai Y, Li B, Lan T, Lai E, et al. STING mediates hepatocyte pyroptosis in liver fibrosis by epigenetically activating the NLRP3 inflammasome. Redox Biol. 2023;62:102691. 37018971 10.1016/j.redox.2023.102691 PMC10106968

[47] Shahzad K , Fatima S, Khawaja H, Elwakiel A, Gadi I, Ambreen S, et al. Podocyte-specific Nlrp3 inflammasome activation promotes diabetic kidney disease. Kidney Int. 2022;102:766–79. 35779608 10.1016/j.kint.2022.06.010

[48] Hsu CG , Li W, Sowden M, Chávez CL, Berk BC. Pnpt1 mediates NLRP3 inflammasome activation by MAVS and metabolic reprogramming in macrophages. Cellular & molecular immunology. 2023;20:131–42. 36596874 10.1038/s41423-022-00962-2 PMC9886977

[49] Kunte SC , Marschner JA, Klaus M, Honda T, Li C, Motrapu M, et al. No NLRP3 inflammasome activity in kidney epithelial cells, not even when the NLRP3-A350V muckle-wells variant is expressed in podocytes of diabetic mice. Front Immunol. 2023;14:14 1230050. 10.3389/fimmu.2023.1230050 PMC10513077 37744356

[50] Swanson KV , Deng M, Ting JP. The NLRP3 inflammasome: molecular activation and regulation to therapeutics. Nat Rev Immunol. 2019;19:477–89. 31036962 10.1038/s41577-019-0165-0 PMC7807242

[51] Shiohara M , Taniguchi S, Masumoto J, Yasui K, Koike K, Komiyama A, et al. ASC, which is composed of a PYD and a CARD, is up-regulated by inflammation and apoptosis in human neutrophils. Biochem Biophys Res Commun. 2002;293:1314–8. 12054656 10.1016/S0006-291X(02)00384-4

[52] Bauernfeind FG , Horvath G, Stutz A, Alnemri ES, MacDonald K, Speert D, et al. Cutting edge: NF-kappaB activating pattern recognition and cytokine receptors license NLRP3 inflammasome activation by regulating NLRP3 expression. J Immunol (Baltimore, Md: 1950). 2009;183:787–91. 19570822 10.4049/jimmunol.0901363 PMC2824855

[53] Mosser DM , Zhang X. Activation of murine macrophages. Curr Protoc Immunol. 2008;83:Chapter 14, pp. 14.12.11-14.12.18. 10.1002/0471142735.im1402s83. PMC2822273 19016446

[54] Yang Y , Wang H, Kouadir M, Song H, Shi F. Recent advances in the mechanisms of NLRP3 inflammasome activation and its inhibitors. Cell Death Dis. 2019;10:128. 30755589 10.1038/s41419-019-1413-8 PMC6372664

[55] Gritsenko A , Yu S, Martin-Sanchez F, Diaz-Del-Olmo I, Nichols EM, Davis DM, et al. Priming is dispensable for NLRP3 Inflammasome activation in human monocytes In vitro. Front Immunol. 2020;11:565924. 33101286 10.3389/fimmu.2020.565924 PMC7555430

[56] Ohto U , Kamitsukasa Y, Ishida H, Zhang Z, Murakami K, Hirama C, et al. Structural basis for the oligomerization-mediated regulation of NLRP3 inflammasome activation. Proc Natl Acad Sci USA. 2022;119:e2121353119. 10.1073/pnas.2121353119. 35254907 PMC8931350

[57] Swanton T , Beswick JA, Hammadi H, Morris L, Williams D, de Cesco S, et al. Selective inhibition of the K(+) efflux sensitive NLRP3 pathway by Cl(−) channel modulation. Chem Sci. 2020;11:11720–8. 34094411 10.1039/d0sc03828h PMC8162947

[58] Xie H , Peng J, Zhang X, Deng L, Ding Y, Zuo X, et al. Effects of mitochondrial reactive oxygen species-induced NLRP3 inflammasome activation on trichloroethylene-mediated kidney immune injury. Ecotoxicol Environ Saf. 2022;244:114067. 36087465 10.1016/j.ecoenv.2022.114067

[59] Gong Z , Pan J, Shen Q, Li M, Peng Y. Mitochondrial dysfunction induces NLRP3 inflammasome activation during cerebral ischemia/reperfusion injury. J Neuroinflammation. 2018;15:242. 30153825 10.1186/s12974-018-1282-6 PMC6114292

[60] Shi H , Wang Y, Li X, Zhan X, Tang M, Fina M, et al. NLRP3 activation and mitosis are mutually exclusive events coordinated by NEK7, a new inflammasome component. Nat Immunol. 2016;17:250–8. 26642356 10.1038/ni.3333 PMC4862588

[61] Li S , Sun Y, Song M, Song Y, Fang Y, Zhang Q, et al. NLRP3/caspase-1/GSDMD-mediated pyroptosis exerts a crucial role in astrocyte pathological injury in mouse model of depression. JCI Insight. 2021;6:e146852. 10.1172/jci.insight.146852. PMC8675200 34877938

[62] Lu Y , Sun Y, Xu K, Saaoud F, Shao Y, Ct D, et al. Aorta in pathologies may function as an immune organ by upregulating Secretomes for immune and vascular cell activation, differentiation and trans-differentiation-early Secretomes may serve as drivers for trained immunity. Front Immunol. 2022;13:13 858256. 10.3389/fimmu.2022.858256 PMC8934864 35320939

[63] Yeung K , Mraz V, Geisler C, Skov L, Bonefeld CM. The role of interleukin-1β in the immune response to contact allergens. Contact Derm. 2021;85:387–97. 10.1111/cod.13955 34324721

[64] Sun Z , Liu H, Hu Y, Luo G, Yuan Z, Tu B, et al. STING contributes to trauma-induced heterotopic ossification through NLRP3-dependent macrophage pyroptosis. Clinical immunology (Orlando, Fla). 2023;250:109300. 36963448 10.1016/j.clim.2023.109300

[65] Ito H , Kanbe A, Sakai H, Seishima M. Activation of NLRP3 signalling accelerates skin wound healing. Exp Dermatol. 2018;27:80–6. 28887870 10.1111/exd.13441

[66] Shi J , Tang Y, Liang F, Liu L, Liang N, Yang X, et al. NLRP3 inflammasome contributes to endotoxin-induced coagulation. Thromb Res. 2022;214:8. 35421682 10.1016/j.thromres.2022.04.001

[67] Boldeanu L , Boldeanu MV, Bogdan M, Meca AD, Coman CG, Buca BR, et al. Immunological approaches and therapy in burns (review). Experimental and therapeutic medicine. 2020;20:2361–7. 32765715 10.3892/etm.2020.8932 PMC7401720

[68] Stanojcic M , Vinaik R, Abdullahi A, Chen P, Jeschke MG. NLRP3 knockout enhances immune infiltration and inflammatory responses and improves survival in a burn sepsis model. Immunology. 2022;165:195–205. 34773253 10.1111/imm.13427 PMC10355283

[69] Liu D , Yang P, Gao M, Yu T, Shi Y, Zhang M, et al. NLRP3 activation induced by neutrophil extracellular traps sustains inflammatory response in the diabetic wound. Clinical science (London, England: 1979). 2019;133:565–82. 30626731 10.1042/CS20180600

[70] Xiao M , Li L, Li C, Liu L, Yu Y, Ma L. 3,4-Methylenedioxy-β-Nitrostyrene ameliorates experimental burn wound progression by inhibiting the NLRP3 Inflammasome activation. Plast Reconstr Surg. 2016;137:566e–75. 10.1097/01.prs.0000479972.06934.83 26910701

[71] Osuka A , Hanschen M, Stoecklein V, Lederer JA. A protective role for inflammasome activation following injury. Shock (Augusta, Ga). 2012;37:47–55. 21921832 10.1097/SHK.0b013e318234f7ff PMC3241872

[72] Stanojcic M , Chen P, Harrison RA, Wang V, Antonyshyn J, Zúñiga-Pflücker JC, et al. Leukocyte infiltration and activation of the NLRP3 inflammasome in white adipose tissue following thermal injury. Crit Care Med. 2014;42:1357–64. 24584061 10.1097/CCM.0000000000000209 PMC4166573

[73] Louiselle AE , Niemiec SM, Zgheib C, Liechty KW. Macrophage polarization and diabetic wound healing. Translational research: the journal of laboratory and clinical medicine. 2021;236:109. 34089902 10.1016/j.trsl.2021.05.006

[74] Diao L , Patsouris D, Sadri AR, Dai X, Amini-Nik S, Jeschke MG. Alternative mechanism for white adipose tissue lipolysis after thermal injury. Molecular medicine (Cambridge, Mass). 2016;21:959–68. 26736177 10.2119/molmed.2015.00123 PMC4818253

[75] Mirza RE , Fang MM, Weinheimer-Haus EM, Ennis WJ, Koh TJ. Sustained inflammasome activity in macrophages impairs wound healing in type 2 diabetic humans and mice. Diabetes. 2014;63:1103–14. 24194505 10.2337/db13-0927 PMC3931398

[76] Wan L , Bai X, Zhou Q, Chen C, Wang H, Liu T, et al. The advanced glycation end-products (AGEs)/ROS/NLRP3 inflammasome axis contributes to delayed diabetic corneal wound healing and nerve regeneration. Int J Biol Sci. 2022;18:809–25. 35002527 10.7150/ijbs.63219 PMC8741862

[77] Gauglitz GG , Herndon DN, Kulp GA, Meyer WJ, 3rd, Jeschke MG. Abnormal insulin sensitivity persists up to three years in pediatric patients post-burn. J Clin Endocrinol Metab. 2009;94:1656–64. 19240154 10.1210/jc.2008-1947 PMC2684478

[78] Gomez R , Murray CK, Hospenthal DR, Cancio LC, Renz EM, Holcomb JB, et al. Causes of mortality by autopsy findings of combat casualties and civilian patients admitted to a burn unit. J Am Coll Surg. 2009;208:348–54. 19317995 10.1016/j.jamcollsurg.2008.11.012

[79] Silva L , Garcia L, Oliveira B, Tanita M, Festti J, Cardoso L, et al. Acute respiratory distress syndrome in burn patients: incidence and risk factor analysis. Annals of burns and fire disasters. 2016;29:178–82. 28149245 PMC5266233

[80] Foncerrada G , Culnan DM, Capek KD, González-Trejo S, Cambiaso-Daniel J, Woodson LC, et al. Inhalation injury in the burned patient. Ann Plast Surg. 2018;80:S98–s105. 29461292 10.1097/SAP.0000000000001377 PMC5825291

[81] Bai C , Li T, Sun Q, Xin Q, Xu T, Yu J, et al. Protective effect of baicalin against severe burn-induced remote acute lung injury in rats. Mol Med Rep. 2018;17:2689–94. 29207058 10.3892/mmr.2017.8120

[82] Han S , Cai W, Yang X, Jia Y, Zheng Z, Wang H, et al. ROS-mediated NLRP3 Inflammasome activity is essential for burn-induced acute lung injury. Mediat Inflamm. 2015;2015:1–16. 10.1155/2015/720457 PMC4630408 26576075

[83] Luo N , Lin H, Zhang L, Jiang Y, Zhao Y, Han Q, et al. Effect of hydrogen on AM Pyroptosis induced by severe burns in rats. Journal of personalized medicine. 2023;13:377. 10.3390/jpm13030377. PMC10053548 36983559

[84] Xiao MJ , Zou XF, Li B, Li BL, Wu SJ, Zhang B. Simulated aeromedical evacuation exacerbates burn induced lung injury: targeting mitochondrial DNA for reversal. Military Medical Research. 2021;8:30. 33985568 10.1186/s40779-021-00320-9 PMC8117593

[85] Vinaik R , Barayan D, Auger C, Abdullahi A, Jeschke MG. Regulation of glycolysis and the Warburg effect in wound healing. JCI Insight. 2020;5:e138949. 10.1172/jci.insight.138949. PMC7526439 32750036

[86] Gauglitz GG , Korting HC, Pavicic T, Ruzicka T, Jeschke MG. Hypertrophic scarring and keloids: pathomechanisms and current and emerging treatment strategies. Molecular medicine (Cambridge, Mass). 2011;17:113–25. 20927486 10.2119/molmed.2009.00153 PMC3022978

[87] Russo S , Kwiatkowski M, Govorukhina N, Bischoff R, Melgert BN. Meta-inflammation and metabolic reprogramming of macrophages in diabetes and obesity: the importance of metabolites. Front Immunol. 2021;12:746151. 34804028 10.3389/fimmu.2021.746151 PMC8602812

[88] Cavalcante-Silva J , Koh TJ. Targeting the NOD-like receptor pyrin domain containing 3 Inflammasome to improve healing of diabetic wounds. Adv Wound Care (New Rochelle). 2022;12:644–56. 34841901 10.1089/wound.2021.0148 PMC10701516

[89] Chi Y , Chai J, Xu C, Luo H, Zhang Q. Apelin inhibits the activation of the nucleotide-binding domain and the leucine-rich, repeat-containing family, pyrin-containing 3 (NLRP3) inflammasome and ameliorates insulin resistance in severely burned rats. Surgery. 2015;157:1142–52. 25817096 10.1016/j.surg.2015.01.011

[90] Deuis JR , Yin K, Cooper MA, Schroder K, Vetter I. Role of the NLRP3 inflammasome in a model of acute burn-induced pain. Burns: journal of the International Society for Burn Injuries. 2017;43:304–9. 28040362 10.1016/j.burns.2016.09.001

[91] Lee JS , Robertson AAB, Cooper MA, Khosrotehrani K. The small molecule NLRP3 Inflammasome inhibitor MCC950 does not Alter wound healing in obese mice. Int J Mol Sci. 2018;19:3289. 10.3390/ijms19113289. PMC6274704 30360489

[92] Bian F , Xiao Y, Zaheer M, Volpe EA, Pflugfelder SC, Li DQ, et al. Inhibition of NLRP3 Inflammasome pathway by butyrate improves corneal wound healing in corneal alkali burn. Int J Mol Sci. 2017;18:562. 10.3390/ijms18030562. PMC5372578 28273882

[93] Vande, Walle L , Stowe IB, Šácha P, Lee BL, Demon D, Fossoul A, et al. MCC950/CRID3 potently targets the NACHT domain of wild-type NLRP3 but not disease-associated mutants for inflammasome inhibition. PLoS Biol. 2019;17:e3000354. 10.1371/journal.pbio.3000354. 31525186 PMC6762198

[94] Zeng W , Wu D, Sun Y, Suo Y, Yu Q, Zeng M, et al. The selective NLRP3 inhibitor MCC950 hinders atherosclerosis development by attenuating inflammation and pyroptosis in macrophages. Sci Rep. 2021;11:19305. 34588488 10.1038/s41598-021-98437-3 PMC8481539

[95] Coll RC , Robertson AA, Chae JJ, Higgins SC, Muñoz-Planillo R, Inserra MC, et al. A small-molecule inhibitor of the NLRP3 inflammasome for the treatment of inflammatory diseases. Nat Med. 2015;21:248–55. 25686105 10.1038/nm.3806 PMC4392179

[96] Wang Y , Wan L, Zhang Z, Li J, Qu M, Zhou Q. Topical calcitriol application promotes diabetic corneal wound healing and reinnervation through inhibiting NLRP3 inflammasome activation. Exp Eye Res. 2021;209:108668. 34144035 10.1016/j.exer.2021.108668

[97] Guo L , Wang Z, Zhu C, Li J, Cui L, Dong J, et al. MCC950 inhibits the inflammatory response and excessive proliferation of canine corneal stromal cells induced by staphylococcus pseudintermedius. Mol Immunol. 2022;152:162. 36370586 10.1016/j.molimm.2022.11.001

[98] Kolterman OG . Glyburide in non-insulin-dependent diabetes: an update. Clin Ther. 1992;14:196–213. 1611644

[99] Shao H , Huang L, Duan S, Gao M, Zhu J, Chen X, et al. Glyburide attenuates ozone-induced pulmonary inflammation and injury by blocking the NLRP3 inflammasome. Environ Toxicol. 2020;35:831–9. 32167222 10.1002/tox.22919

[100] Lamkanfi M , Mueller JL, Vitari AC, Misaghi S, Fedorova A, Deshayes K, et al. Glyburide inhibits the Cryopyrin/Nalp3 inflammasome. J Cell Biol. 2009;187:61–70. 19805629 10.1083/jcb.200903124 PMC2762099

[101] Wang Y , Zhang XL, Sun CM. BAY-11-7082 induces apoptosis of multiple myeloma U266 cells through inhibiting NF-κB pathway. Eur Rev Med Pharmacol Sci. 2018;22:2564–71. 29771406 10.26355/eurrev_201805_14949

[102] Strickson S , Campbell DG, Emmerich CH, Knebel A, Plater L, Ritorto MS, et al. The anti-inflammatory drug BAY 11-7082 suppresses the MyD88-dependent signalling network by targeting the ubiquitin system. The Biochemical journal. 2013;451:427–37. 23441730 10.1042/BJ20121651 PMC3685219

[103] Irrera N , Vaccaro M, Bitto A, Pallio G, Pizzino G, Lentini M, et al. BAY 11-7082 inhibits the NF-κB and NLRP3 inflammasome pathways and protects against IMQ-induced psoriasis. Clinical science. London, England: 1979. 2017;131:487–98. 10.1042/CS20160645 28096316

[104] Jiang W , Li M, He F, Zhou S, Zhu L. Targeting the NLRP3 inflammasome to attenuate spinal cord injury in mice. J Neuroinflammation. 2017;14:207. 29070054 10.1186/s12974-017-0980-9 PMC5657095

[105] Bitto A , Altavilla D, Pizzino G, Irrera N, Pallio G, Colonna MR, et al. Inhibition of inflammasome activation improves the impaired pattern of healing in genetically diabetic mice. Br J Pharmacol. 2014;171:2300–7. 24329484 10.1111/bph.12557 PMC3997271

[106] Messerschmitt PJ , Rettew AN, Schroeder NO, Brookover RE, Jakatdar AP, Getty PJ, et al. Osteosarcoma phenotype is inhibited by 3,4-Methylenedioxy-β-nitrostyrene. Sarcoma. 2012;2012:1–11. 10.1155/2012/479712 PMC3371351 22701331

[107] Wang WY , Hsieh PW, Wu YC, Wu CC. Synthesis and pharmacological evaluation of novel beta-nitrostyrene derivatives as tyrosine kinase inhibitors with potent antiplatelet activity. Biochem Pharmacol. 2007;74:601–11. 17601492 10.1016/j.bcp.2007.06.001

[108] He Y , Varadarajan S, Muñoz-Planillo R, Burberry A, Nakamura Y, Núñez G. 3,4-methylenedioxy-β-nitrostyrene inhibits NLRP3 inflammasome activation by blocking assembly of the inflammasome. J Biol Chem. 2014;289:1142–50. 24265316 10.1074/jbc.M113.515080 PMC3887181

[109] Long H , Xu B, Luo Y, Luo K. Artemisinin protects mice against burn sepsis through inhibiting NLRP3 inflammasome activation. Am J Emerg Med. 2016;34:772–7. 26830216 10.1016/j.ajem.2015.12.075

